# Air Quality in the Working Environment and Respiratory Health of Female Congolese Stone Quarry Workers

**DOI:** 10.3390/tropicalmed5040171

**Published:** 2020-11-17

**Authors:** Ngombe Leon-Kabamba, Nlandu Roger Ngatu, Basilua Andre Muzembo, Sakatolo Kakoma, Nzaji Michel-Kabamba, Brigitta Danuser, Oscar Luboya, Tomohiro Hirao

**Affiliations:** 1 Department of Public Health, University of Kamina, Kamina, Congo; michelnzaji@yahoo.fr (N.M.-K.); oscarluboya@yahoo.fr (O.L.); 2 Technical Medical College of Lubumbashi (ISTM-Lubumbashi), Lubumbashi, Congo; 3 Department of Public Health, Kagawa University Faculty of Medicine, Miki-cho 761-0793, Japan; sharks@med.kagawa-u.ac.jp; 4 Graduate School of Dentistry and Pharmaceutical Sciences, Okayama University, Okayama 700-8558, Japan; andersonbasilua@yahoo.fr or; 5 School of Public Health, University of Lubumbashi, Lubumbashi, Congo; jbszkakoma2016@gmail.com; 6 Institute for Work and Health Service, University of Lausanne and Geneva, CH-1011 Lausanne, Switzerland; Brigitta.Danuser@chuv.ch

**Keywords:** air quality, Congo, PM_2.5_, quarry worker, respiratory health, working environment

## Abstract

Background and Aim. Environmental and occupational exposure to high dust levels are known to be associated with lung function impairment. We assessed the ambient air quality in the working environment and the respiratory health of female stone quarry workers in Lubumbashi, Democratic Republic of Congo (DRC) in a context of severe economic, security, and health crises. Methods. This was a case-control study conducted in three stone quarry sites. Participants were 256 dust-exposed female stone quarry workers matched to 256 unexposed female office workers and market tax collectors (*N =* 512). They each answered a structured respiratory health questionnaire and underwent physical examination and a lung function test with the use of a spirometer and peak flow meter. Quality of ambient air in the working environment was assessed by means of a BRAMC air quality monitor (BR-AIR-329). Results. Results showed that exposed women did not use any personal protective equipment (PPE); in quarry sites, abnormally high levels of PM_2.5_ (205 *±* 13.2 μg/m^3^ vs. 31.3 *±* 10.3 μg/m^3^ in control sites; *p <* 0.001) and volatile organic compounds (VOC, 2.2 *±* 0.2 μg/m^3^ vs. 0.5 *±* 0.3 μg/m^3^, respectively; *p <* 0.01) were found. Furthermore, respiratory complaints were more common among exposed women (32.4% vs. 3.5% in controls; *p <* 0.01), who had abnormal chest auscultation and reduced lung capacity than controls (mean PEFR: 344.8 *±* 2.26 and 405 *±* 67.7 L/s, respectively; *p <* 0.001 Conclusion. Findings from this study show that in the midst of severe crises in the DRC, women stone quarry workers are exposed to abnormally high levels of respiratory hazards, which contribute to impaired lung function. There is a need to regulate quarry work and improve the working conditions in quarry sites in the DRC.

## 1. Introduction

Air pollution poses a major threat to health, causing one in nine deaths. The United Nations (UN) Environment Report 2017 has stated that air pollution is the most important environmental health risk of our time [1,2]. Air pollution can occur both in the living and working environments, and the association between environmental and occupational exposure to fine particles such as PM_2.5_ and other ultrafine particulate matter and the development of chronic lung disorders is well established [1,2,3]. Considering stone quarry workers, previous reports have shown an association between occupational dust-exposure and impaired lung function [2].

Though there are several sources of air pollutants with an impact on the respiratory health of exposed workers (power plants, cement factories, construction industries, petrochemical industries, mining, etc.), the emissions of particulates from quarries are quite high [3,4,5,6,7]. Individuals exposed to high particulate matter (PM_2.5_ in particular) levels at their working or living environments are at a higher risk of developing a number of respiratory disorders, such as chronic airway inflammatory diseases (bronchitis, emphysema, silicosis) and lung cancer [6,7].

Furthermore, the health impacts of working in stone quarry settings are well documented [4,5]. For instance, epidemiological studies have supported the association between respiratory impairment and occupational exposure to dust. Additionally, individuals working in dusty environments have been found to carry the risk of inhaling particulate materials (e.g., silica) that may lead to adverse respiratory effects, such as chronic bronchitis, emphysema, acute and chronic silicosis, and lung cancer, which are disabling and can even be fatal [5,7]. Through its global development agenda, the United Nations’ Sustainable Development Goals (SDGs) are calling to end poverty (SDG1), and promote “decent work and economic growth” (SDG8) and health for all (SDG3) as well. These goals cannot be reached without the provision of decent and satisfactory working conditions and environments to the employees, particularly women [8]. Studies on occupational safety and health of African quarry workers are scarcely found in the literature.

A few studies conducted in Nigeria showed that quarry workers with a history of chronic dust exposure had a higher risk of developing respiratory symptoms [9,10]; however, there have been no study on women, particularly those from countries of the central African region. Considering the poor working conditions in Congolese informal quarry sites, we hypothesized that more than half of the exposed women stone quarry workers would report respiratory manifestations occurring in the previous 12 months.

The aim of the present study was to determine the ambient levels of PM_2.5_ and volatile organic compounds (VOC) in the working environment, and to investigate the respiratory health status of female Congolese stone quarry workers in Lubumbashi, in the Democratic Republic of Congo (DRC). Its findings would allow the researchers to issue a warning to the employers and health policy-makers in the DRC on the impact of dust on those women’s health and enable the implementation of preventive measures.

## 2. Materials and Methods

### 2.1. Study Design, Sites, and Participants

**Figure 1:**
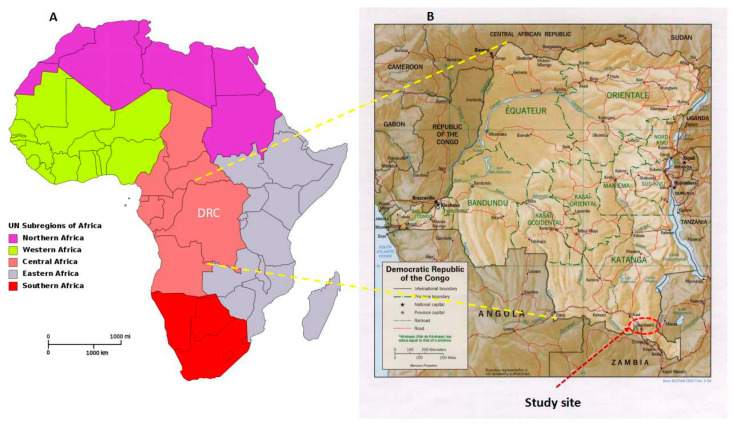
Map of Africa (**A**) and the Democratic Republic of Congo (**B**) with study sites located in Lubumbashi, southern province of Haut-Katanga, in the Democratic Republic of Congo (source of maps: Wikimedia commons).

This was a case-controlled study conducted in the city of Lubumbashi, Haut-Katanga province [11], in the DRC (Figure 1), from 1 May 2016 through July 2017. Katanga, a territory that is part of the African copper-belt, is the second largest town in the DRC and is considered as the economic capital of the country due to its rich mining industries.

**Figure 2:**
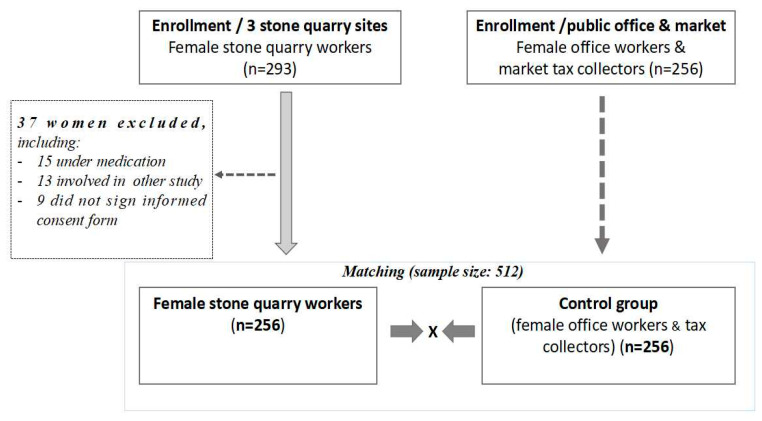
Diagram showing the sampling process of study participants.

All 293 women who have been working at three informal stone quarry sites located in Lubumbashi were enrolled in this study. Of those female stone quarry workers, 256 (exposed group) who met the inclusion criteria were age-matched to 256 female office workers and market tax collectors (control group) (*N =* 512) (Figure 2). Of the 293 enrolled stone quarry workers, 37 were excluded as they did not meet the inclusion criteria. Controls were public administrative office workers and market tax collectors without known history of obvious occupational or environmental exposure to dust.

Inclusion criteria for the exposed women were as follows: voluntary participation; having at least one year of work experience; absence of spirometry contraindication; not being under steroid, bronchodilator, or anti-tuberculosis therapy; and not participating in a similar study. The 37 female stone quarry workers who were excluded comprised 15 women who were receiving either a steroid therapy or a bronchodilator-based treatment; 13 were involved in another study during the same period, and 9 declined to sign informed consent forms.

### 2.2. Survey Questionnaire and Outcome Variables

The survey and lung function testing took place at the work sites of the participants. The medical check-up consisted of medical and occupational history-taking as well as lung auscultation. Participants answered a structured respiratory health survey questionnaire [12], which comprised questions related to anthropometric, sociodemographic, and occupational characteristics, as well as a respiratory health history. A lung function test was performed with the use of a spirometer, according to the ATS/ERS 2005 protocol, and a peak flow meter for the determination of peak expiratory flow rate (PEFR). Each subject had to undergo three consecutive tests, and the best result was considered. The following lung function parameters were considered in the data analysis: forced vital capacity (FVC), forced expiratory volume per second (FEV1), and PEFR.

Work site ambient air quality assessment was performed at different work stations within both the stone quarry sites and the control sites to determine PM_2.5_ and volatile organic compound (VOC) concentrations, as reported previously [12]. Air dust level measurements were carried out three times at each of three randomly selected work stations at each work site, with a 30-min interval during working time, by means of a BRAMC air quality monitor (BR-AIR-329; Shandong, China). The mean PM_2.5_ and VOC values were calculated and considered in this study.

### 2.3. Ethical Considerations, Data Collection, and Statistical Analysis

Participation in this study was voluntary, and each participant provided written informed consent prior to her enrollment. All clinical procedures were performed in conformity with internationally accepted regulations in regard to the use of human subjects in research. Ethical approval (UNILU/CEM/075/2015) was granted by the ethics committee of the School of Public Health, University of Lubumbashi, DRC. Survey data were collected and then transcribed in a prepared Excel sheet. For continuous variables such as lung function parameters, group comparison was carried out using Student’s *t*-test, whereas Fisher’s exact test was performed for categorical or dichotomized variables. To determine the relationship between the characteristics of the participants and the reported respiratory manifestations, a bivariate analysis was performed with the use of a logistic regression test. Additionally, factors that showed associations in the bivariate analysis were entered in a multivariate logistic regression model. A *p*-value (double-sided) less than 0.05 was considered significant. Stata statistical software version 14 (Stata Corporation, College Station, TX, USA) was used for the analyses.

## 3. Results

### 3.1. Air Quality in the Working Environment and Characteristics of the Participants

Dust emissions were observed at the stone quarry work sites, and none of the exposed women had been using any protective device (mask) to prevent dust inhalation. Workplace air dust measurements showed higher PM2.5 (205 *±* 13.2 μg/m^3^ vs. 31.3 *±* 10.3 μg/m^3^; *p <* 0.001) and VOC (2.2 *±* 0.2 μg/m^3^ vs. 0.5 *±* 0.3 mg/m^3^; *p <* 0.01) levels at quarry work sites than in control sites (not shown).

Table 1 shows the characteristics of the study participants. Almost all exposed women (254/256; 99.2%) were nonsmokers; only two of them (0.8% vs. 3.5% in controls; *p <* 0.05) were smoking at the time of this study. The exposed women worked longer than controls, 12.0 *±* 0.0 h a day versus 9.4 *±* 1.4 h (*p <* 0.001), respectively. In addition, a higher proportion of control women had either completed the high school level or higher level (23.1% vs. 4.3%; *p <* 0.01). Furthermore, a higher proportion of exposed women had an abnormal auscultation outcome (rales, rhonchi, or wheeze) as compared with control women (32.4% vs. 3.5%; *p <* 0.01) (Table 1).

### 3.2. Prevalence of Respiratory Complaints Among the Exposed Workers and Controls, and Lung Function Outcomes

Prevalence of respiratory complaints and disorders was higher in women stone quarry workers than in controls: wheezing (27% vs. 9.1%; *p <* 0.001), wheezing after effort (22.3% vs. 9.1%; *p <* 0.05), morning cough (51.6% vs. 3.2%; *p <* 0.001), shortness of breath at rest (31.3% vs. 1.1%; *p <* 0.001), shortness of breath after effort rest (38.7% vs. 0.5%; *p <* 0.001), morning phlegm (41% vs. 1.6%; *p <* 0.001), chronic bronchitis (17.6% vs. 1.1%; *p <* 0.001), and rhinitis (57.8% vs. 13.4%; *p <* 0.001) (Table 2).

**Figure 3:**
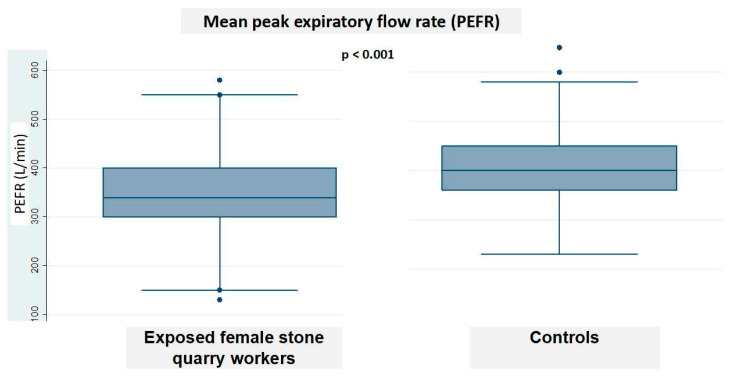
Box plot image showing the distribution of mean PEFR values in exposed women stone quarry workers and controls.

Figure 3 shows the outcomes of the lung function test. A significantly lower mean PEFR (344.8 *±* 2.26 vs. 405 *±* 67.7 L/s; *p <* 0.001) was observed in the dust-exposed stone quarry workers as compared with controls. Regarding spirometry outcomes, reduced lung volumes were observed in the group of exposed female stone quarry workers compared to the control for FVC (3.6 *±* 0.7 vs. 4.3 *±* 0.9 L/s) and FEV1 (3.1 *±* 0.7 vs. 3.4 *±* 0.7 L/s), but not significantly (not shown).

### 3.3. Association Between Occupational Characteristics and Respiratory Manifestations

Multivariate logistic regression analysis was performed to assess the association between respiratory complaints and quarry work. It was observed that most complaints were positively associated with stone quarry work, except asthma. After adjusting for age, education, and smoking, most of the respiratory complaints and disorders remained associated with quarry work as occupation: wheezing at rest (aOR = 6.35 *±* 2.61; 95%CI: 2.61–15.43; *p* < 0.001), wheezing after effort (aOR = 3.36 *±* 1.64; 95%CI: 1.28–8.78; *p* = 0.013), night cough (aOR = 0.65 *±* 0.23; 95%CI: 0.32–1.31; *p =* 0.234), morning cough (aOR = 6.06 *±* 1.88; 95%CI: 3.29–11.15; *p <* 0.001), breathlessness/rest (aOR = 3.95 *±* 1.15; 95CI%: 2.22–7.02; *p <* 0.001), breathlessness/effort (aOR = 5.16 *±* 1.41; 95%CI: 2.46–10.84; *p <* 0.001), morning phlegm (aOR = 2.62 *±* 0.99; 95CI%: 1.24–5.52; *p <* 0.001), chronic bronchitis (aOR = 2.61 *±* 0.75; CI 95%: 1.48–4.61; *p <* 0.001), asthma (aOR = 0.36 *±* 0.29; 95%CI: 0.07–1.77; *p* = 0.211), and rhinitis (aOR = 2.13 *±* 0.21; 95%CI: 1.18–3.39; *p <* 0.001) (Table 3).

## 4. Discussion

This work was the first study conducted in the female stone quarry workers in Africa. It was observed that female stone quarry workers had very low socioeconomic status and education level. Additionally, despite workplace dust (PM_2.5_, VOC) concentrations beyond currently accepted exposure limits [13,14], these women did not use either personal protective equipment (PPE) against dust or recommended general measures to reduce dust levels in occupational settings. Similar findings have been previously reported [15,16].

This study also showed that dust-exposed female stone quarry workers reported more respiratory complaints and disorders than did the controls, with reduced lung function (PEFR) and higher proportion (32.4%) of exposed women with abnormal auscultation, as compared with controls. Rates of almost all respiratory complaints found in our study were higher than those reported among Iranian, Indian, and Nigerian quarry and cement factory workers [15,16,17,18,19]. Female stone quarry workers from our study were more likely to report morning phlegm, morning cough, and chronic rhinitis than quarry workers at a stone crushing industrial site in Nigeria [10], whereas the decline in PEFR value was similar to that observed among dust-exposed Nigerian cement factory workers [20,21]. This suggests that dust-exposure in quarry sites represents an important occupational health hazard in Lubumbashi, DRC.

PM_2.5_ is one of the main causes of COPD and an important environmental health risk factor that can induce lung function impairment in exposed individuals [21,22,23,24,25]. Our study showed PM_2.5_ levels above the occupational exposure limit (OEL) of 25 μg/mL per 24 h recommended by the World Health Organization (WHO) [25,26], suggesting that the high proportion of respiratory manifestations observed among female stone quarry workers in our study, and the consecutive reduction of lung function could be explained, at least partially, by the long-term dust-exposure.

Furthermore, our study showed an inverse correlation between PEFR and PM_2.5_ level, and between PEFR and working hours as well in female quarry workers. These outcomes suggest that chronic exposure to dust at stone quarry work sites and daily exposure duration could be considered predisposing factors for respiratory disorders and the decline of lung function in this category of workers. Similar findings have been reported previously [12,25,26].

Smoking is well established as a cause and trigger of respiratory disorders. In our study, no correlation was observed between smoking and lung function status (PEFR). This can be explained by the fact that almost all (99.2%) exposed women were nonsmokers, suggesting that the lung function decline and respiratory complaints reported by those women might have been induced by dust-exposure at work. Another strong point of this study is the relatively high sample size and the methodology used in enrolling participants. For example, exposed and control women were all women, age-matched, and shared the same living environment. Moreover, this study is the first to report on the effects of chronic exposure to mineral dust in a particularly vulnerable category of the Congolese population (women) that is still experiencing one of the most severe crises in the world, namely extreme poverty and severe gender-based human rights issues [27,28].

This study is limited by its cross-sectional design, which implies the difficulty to generalize its outcomes, and the fact that only work site area dust levels were measured but not personal exposure levels. Nonetheless, in addition to the respiratory health survey, the sample size was relatively large, and objective investigations such as physical examination and lung function tests were performed to determine the impact of occupational dust-exposure to the respiratory health of the exposed women. Furthermore, in this study, a control group was used, which comprised local women administrative workers having no evident occupational dust-exposure history. Thus, the respiratory defect observed among the exposed stone quarry workers might have been solely caused by dust of occupational origin.

In conclusion, the present study highlights the poor and hazardous working environment of women stone quarry workers in the Congo, with a high risk of developing respiratory manifestations. As a recommendation, there is a necessity to regulate informal activities related to the construction sector in DRC and promote occupational safety and health for the workers. In addition, health policy makers in DRC should recognize informal quarry work as hazardous for the health of workers and the use of efficient preventive measures against dust should be compulsory to protect the health of exposed women.

## Figures and Tables

**Table 1:** Sociodemographic and clinical characteristics of participants.

Sociodemographic and Clinical Characteristics	Exposed Female Quarry Workers (*N* = 256)	Controls (n = 256)	*p*-Value
	Mean *±* SD	Mean *±* SD	
Age (years)	43.26 *±* 10.82	44.07 *±* 9.41	0.38
Working years	2.92 *±* 4.55	8.35 *±* 7.06	<0.001
Daily work duration (hours)	12 *±* 0.00	9.41 *±* 1.43	<0.001
	n (%)	n (%)	
Education, primary/secondary	245 (95.7)	197 (76.9)	<0.01
High school or higher	11 (4.3)	59 (23.1)	
Smoking status, Yes	2 (0.8)	9 (3.5)	0.033
No	254 (96.2)	247 (96.5)	
Alcohol intake, Yes	37 (14.5)	31 (12.1)	0.435
No	219 (85.5)	225 (87.9)	
Lung auscultation, Abnormal	83 (32.4)	09 (3.5)	<0.01
Normal	173 (67.6)	247 (96.5)	

Notes: n, sample size; SD, standard deviation.

**Table 2:** Prevalence of respiratory manifestations in quarry workers and controls.

Respiratory Complaints/Disorders	Female Quarry Workers *N* = 256 (%)	Controls *N* = 256 (%)	*p*-Value
Wheezing	69 (27)	17 (9.1)	<0.001
Wheezing/effort	57 (22.3)	17 (9.1)	<0.001
Night cough	64 (25)	23 (12.3)	0.001
Shortness of breath/rest	80 (31.3)	2 (1.1)	<0.001
Shortness of breath/effort	99 (38.7)	1 (0.5)	<0.001
Asthma	6 (2.3)	3 (1.6)	0.43
Morning cough	132 (51.6)	6 (3.2)	<0.001
Morning phlegm	105 (41)	3 (1.6)	<0.001
Chronic bronchitis	45 (17.6)	2 (1.1)	<0.001
Rhinitis	148 (57.8)	25 (13.4)	<0.001

**Table 3:** Association between respiratory manifestations and stone quarry work.

Respiratory Manifestations	Model 1			Model 2		
		95%CI	*p*-Value	aOR (SE)	95%CI	*p*-Value
Wheezing at rest	1.09 (0.26)	0.67–1.76	0.714	-	-	-
Wheezing after effort	2.42 (0.61)	1.47–3.98	<0.001	3.36 (1.64)	1.28–8.78	0.013
Night cough	0.42 (0.11)	0.26–0.68	<0.01	0.65 (0.23)	0.32–1.31	0.234
Morning cough	7.72 (1.76)	4.93–12.09	<0.001	6.06 (1.88)	3.29–11.15	<0.001
Morning phlegm	5.89 (1.41)	3.68–9.43	<0.001	2.62 (0.99)	1.24–5.52	0.011
Breathlessness at rest	8.49 (2.67)	4.58–15.75	<0.001	3.95 (1.15)	2.22–7.02	<0.001
Breathlessness after effort	6.09 (1.52)	3.73–9.94	<0.001	5.16 (1.95)	2.46–10.84	<0.001
Asthma	1.21 (0.73)	0.36–3.99	0.761	-	-	-
Chronic bronchitis	2.66 (0.77)	1.50–4.69	0.001	2.61 (0.75)	1.48–4.61	<0.001
Rhinitis	5.37 (1.07)	3.63–7.96	<0.001	2.13 (0.21)	1.18–3.39	<0.001

**Notes:** OR, odds ratio; aOR, adjusted odds ratio; SE, standard error; 95%CI, 95% confidence interval. Model 1, logistic regression analysis without adjustment; Model 2, logistic regression analysis with adjustment for age, education, and smoking.

## Data Availability

Data and materials related to this study are available at the Department of Public Health, University of Kamina, Democratic Republic of Congo.
